# Effect of Small Molecules Modulating Androgen Receptor (SARMs) in Human Prostate Cancer Models

**DOI:** 10.1371/journal.pone.0062657

**Published:** 2013-05-08

**Authors:** Anna Tesei, Carlo Leonetti, Marzia Di Donato, Elisa Gabucci, Manuela Porru, Greta Varchi, Andrea Guerrini, Dino Amadori, Chiara Arienti, Sara Pignatta, Giulia Paganelli, Michele Caraglia, Gabriella Castoria, Wainer Zoli

**Affiliations:** 1 Biosciences Laboratory, IRCCS Istituto Scientifico Romagnolo per lo Studio e la Cura dei Tumori (IRST), Meldola, Italy; 2 Department of Experimental Oncology, Laboratory of Experimental Preclinical Chemotherapy, National Cancer Institute “Regina Elena”, Rome, Italy; 3 Department of General Pathology, II University of Naples, Naples, Italy; 4 Italian National Research Council, Institute for Organic Chemistry and Photoreactivity, Bologna, Italy; 5 Department of Medical Oncology, IRCCS Istituto Scientifico Romagnolo per lo Studio e la Cura dei Tumori (IRST), Meldola, Italy; 6 Department of Biochemistry and Biophysics, Second University of Naples, Naples, Italy; II Università di Napoli, Italy

## Abstract

The management of hormone-refractory prostate cancer represents a major challenge in the therapy of this tumor, and identification of novel androgen receptor antagonists is needed to render treatment more effective. We analyzed the activity of two novel androgen receptor antagonists, (*S*)-**11** and (*R*)-**9**, in *in vitro* and *in vivo* experimental models of hormone-sensitive or castration-resistant prostate cancer (CRPC). *In vitro* experiments were performed on LNCaP, LNCaP-AR, LNCaP-Rbic and VCaP human prostate cancer cells. Cytotoxic activity was assessed by SRB and BrdU uptake, AR transactivation by luciferase reporter assay and PSA levels by Real Time RT-PCR and ELISA assays. Cell cycle progression-related markers were evaluated by western blot. *In vivo* experiments were performed on SCID mice xenografted with cells with different sensitivity to hormonal treatment. In hormone-sensitive LNCaP and LNCaP-AR cells, the latter expressing high androgen receptor levels, (*R*)-**9** and (*S*)-**11** exhibited a higher cytotoxic effect compared to that of the reference compound ((*R*)-bicalutamide), also in the presence of the synthetic androgen R1881. Furthermore, the cytotoxic effect produced by (*R*)-**9** was higher than that of (*S*)-**11** in the two hormone-resistant LNCaP-AR and VCaP cells. A significant reduction in PSA levels was observed after exposure to both molecules. Moreover, (*S*)-**11** and (*R*)-**9** inhibited DNA synthesis by blocking the androgen-induced increase in cyclin D1 protein levels. *In vivo* studies on the toxicological profile of (*R*)-**9** did not reveal the presence of adverse events. Furthermore, (*R*)-**9** inhibited tumor growth in various *in vivo* models, especially LNCaP-Rbic xenografts, representative of recurrent disease. Our *in vitro* results highlight the antitumor activity of the two novel molecules (*R*)-**9** and (*S*)-**11**, making them a potentially attractive option for the treatment of CRPC.

## Introduction

Prostate cancer is the second most frequently diagnosed cancer and the sixth leading cause of cancer death among men worldwide [1]. In developed countries, including Italy, it is the most common malignancy in men and second only to lung cancer in terms of cancer mortality [2], [3]. Surgery, radiotherapy and/or androgen deprivation are the most effective clinical therapies in the early stages of the disease. In particular, hormonal therapy leads to remission, which typically lasts from 2 to 3 years. Nevertheless, prostate cancer frequently metastasizes to bone and almost invariably progresses to an androgen-independent state, with a poor prognosis and a median survival ranging from 10 to 20 months [4]. To date, much of the research into prostate cancer has been geared towards androgens, focusing mainly on ways of decreasing circulating androgens and of inhibiting androgen receptor (AR) functionality.

Antiandrogens, classified as steroid or non-steroid compounds, inhibit androgen activity by competitively blocking the interaction between testosterone and/or dihydrotestosterone (DHT) and AR. Steroid antiandrogens, among which cyproterone acetate is the most representative, have recently been the subject of much discussion because of their side-effects, similar to those induced by castration therapy [5]. There is also concern about their apparent hepatotoxicity from long-term use and thromboembolic complications caused by their potential progestogenic action [6].

Nonsteroid antiandrogens, such as bicalutamide (Casodex®), flutamide (Eulexin®) and nilutamide (Nilandron®) appear to be better tolerated than their steroid analogs and are currently the only available means of avoiding castration in the endocrine treatment of prostate cancer [5]. These compounds are often referred to as “pure antiandrogens” because they bind exclusively to the AR. Bicalutamide is the best tolerated of these drugs, [7]–[9] but, like the other two, it acts as an agonist when AR mutations occur (W741C e H874Y) and/or in cases of AR overexpression, as occurs in hormone refractory prostate cancer. There is now general consensus about the key role of AR in the etiology and progression of prostate cancer (PC), even when it evolves from androgen-sensitive to castration-resistant disease (CRPC). This is supported by evidence that AR expression is preserved in most prostate cancer specimens, regardless of the stage and grade, and by the fact that only a small proportion of CRCP patients experience loss of AR expression, presumably through AR promoter methylation [10]. Furthermore, studies on tumor samples from CRPC patients have revealed several mechanisms used by tumor cells to reactivate AR signaling, *e.g.* AR amplification or mutation, changes in the expression of enzymes involved in steroidogenesis, and intracrine androgen production [11]–[25]. Despite the clinical benefit of both first- and second-line hormone therapies, the most widely used antiandrogens, including bicalutamide, have low AR affinity [26]. These findings have led to the search for new molecules with higher AR-affinity in order to increase clinical effectiveness.

The present preclinical study aimed to investigate the activity and mechanisms of action of new small organic molecules capable of functioning as androgen receptor antagonists in LNCaP cells, which harbor a mutation at codon 877 of the AR ligand-binding domain [27], and in different cell lines representative of CRPC conditions.

## Materials and Methods

### Drugs and Chemicals

Pure (*R*)-bicalutamide (the active enantiomer of the non steroidal antiandrogen, Casodex®) and compounds (*S*)-**11** and (*R*)-**9** were straightforwardly synthesized by highly diastereoselective procedures (Patent Application no. WO2010/116342A2 and no. WO2010/092546A1 [28]), starting from (D)-malic acid, (D)-phenylglicine and (D)-phenylalanine, respectively. (*S*)-**11** and (*R*)-**9** were dissolved in acetone and ethanol (10 mM), respectively, whereas the synthetic androgen methyltrienolone R1881 (Chemos-GmbH, Regenstauf, Germany) was solubilized in DMSO (35.2 mM) and stored at −20°C until use. Casodex® was purchased from Sigma-Aldrich (Milan, Italy).

### Constructs

cDNA encoding the wild-type hAR was in pSG5 [29]. The 3416 construct, containing four copies of the wild-type slp-HRE2 (59-TGGTCAgccAGTTCT-39), was cloned in the NheI site in pTKTATA-Luc [30].

### Cell Lines and Culture

The human prostate cancer-derived cell lines LNCaP and VCaP were obtained from the American Tissue Culture Collection (Rockville, MD, USA). The LNCaP-AR cell line, derived from LNCaP and engineered to stably express high levels of AR, was kindly provided by Dr. Charles L. Sawyers (Howard Hughes Medical Institute Investigator at Memorial Sloan Kettering Cancer Center in New York, U.S.A). The (*R)*-bicalutamide resistant subclone of LNCaP cells (LNCaP-Rbic) was isolated in our laboratory by culturing LNCaP parental cell line for 50 weeks with 20 µM of (*R*)-bicalutamide.

VCaP cells were maintained in DMEM medium supplemented with 10% fetal calf serum (Mascia Brunelli S.p.A., Milan, Italy). LNCaP and LNCaP-AR cells were maintained in RPMI medium supplemented with 10% fetal calf serum (FCS) and 1% glutamine (Mascia Brunelli S.p.A., Milan, Italy). LNCaP-Rbic cells were maintained in the same way supplemented with (*R*)-bicalutamide. Unless otherwise stated, cells were used in the exponential growth phase in all experiments. Cos 7 cells were grown in DME supplemented with phenol red, 5% fetal calf serum (FCS), insulin (6 ng/ml), l-glutamine (2 mM), penicillin (100 U/ml), streptomycin (100 U/ml) and hydrocortisone (3.75 ng/ml). The cells were made quiescent using phenol red-free DMEM and dextran charcoal-treated calf serum. All these procedures have been described previously [31]–[33].

### Transfection, Nuclear Translocation and Transactivation Assay

For AR translocation analysis (Fig. S5), Cos-7 cells at 70% confluence were transfected with 1 µg of purified plasmid using the Superfect reagent (Qiagen, Hilden, Germany). Cells were then made quiescent and left unstimulated or stimulated with 10 nM R1881 for 60 min in the absence or presence of the study compounds. For the transactivation assay (Fig. S4), Cos-7 cells were plated at 70% confluence in phenol red–free DME containing 5% charcoal-stripped serum. After 48 hours, cells were transfected by Superfect with 0.8 µg of 3416-pTK-TATA-Luc, alone or with 0.2 µg of pSG5-hAR–expressing plasmid. After 18 hours, transfected cells were stimulated with 10 nM R1881 (dissolved in 0.001% ethanol, final concentration) for 24 hours in the absence or presence of the indicated compounds. Control cells were treated with the vehicle alone. Cell lysates were prepared and luciferase activity was measured using a luciferase assay system (Promega). The results were corrected using CH110-expressed-galactosidase activity.

### AR Ligand Binding Displacement Studies in LNCaP Cells

LNCaP cells were maintained as previously reported [32] and made quiescent using phenol red-free medium and dextran charcoal-stripped serum [32]. Cells at 70% confluence were incubated by adding 10 nM of [3H]R1881 (98 Ci/mmole; Perkin Elmer) to the medium in the absence or presence of the indicated excess of radio-inert compounds. After a 4-hour incubation at 37°C, the cells were washed three times with ice-cold PBS and collected by gently scraping in the cold room using 600 µl of ice-cold PBS containing 0.05% EDTA (w/v). The number of cells in an aliquot of 100 µl was counted. An aliquot (200 µl) of cell suspension was submitted in duplicate to the extraction of intracellular radioactivity using 500 µl ice-cold ethanol (100%) for 1 hour [33]. After 24 hours at 37°C, radioactivity was counted in a liquid scintillation counter. Nonspecific binding of [3H] R1881 was determined in separate wells by adding the indicated excess of unlabeled R1881 to the incubation medium.

### In vitro Chemosensitivity Assay

Sulforhodamine B (SRB) assay was used according to the method by Skehan et al. [34]. Briefly, cells were collected by trypsinization, counted and plated at a density of 5,000 cells/well in 96-well flat-bottomed microtiter plates (100 µl of cell suspension/well). Experiments were run in octuplicate and each experiment was repeated three times. The optical density of cells was determined at a wavelength of 490 nm by a colorimetric plate reader. Dose response curves were created by Excel software and 50% inhibiting concentration (IC_50_) values were determined graphically from the plots.

### Quantitative Real-time PCR

Total cellular RNA was isolated using RNeasy Minikit (Qiagen). One microgram of RNA was reverse transcribed into cDNA using iScript (BioRad, Hercules, CA) according to the manufacturer’s instructions. Real-time PCR was performed using the MyiQ Single Color Real-Time PCR Detection System (BioRad) and SYBR Green I dye chemistry. The stably expressed endogenous β2-microglobulin gene was amplified as a control for quality and quantity of input RNA. Primers for mRNA amplification GAPDH, forward 5′-CGCTACTCTCTCTTTCTGGC-3′, reverse 5′-AGACACATAGCAATTCAGAAT-3′; HPRT forward 5′- AGACTTTGCTTTCCTTGGTCAGG -3′, reverse 5′- GTCTGGCTTATATCCAACATTCG -3′; PSA forward 5′- GCAGCATTGAACCAGAGGAG -3′, reverse 5′- CCATGACGTGATACCTTGA -3′) were designed using Beacon Designer Software (Version 7.2, BioRad). Real-time PCR was carried out in triplicate reactions at a final volume of 25 ml containing 50 ng of cDNA template, SYBR Green Mix, and 200 nM of forward and reverse primers. Samples were maintained at 95°C for 10 minutes and 30 seconds, followed by 40 amplification cycles at 95°C for 15 seconds, and then at 60°C for 30 seconds for GAPDH, HPRT and PSA. Product specificity was controlled by melting point analysis. Amplification efficiency, which never varied by >5% in the different experiments, was used to determine the relative expression of mRNA obtained using Gene Expression Macro Software (Version 1.1) (BioRad). The amount of mRNA was normalized to the endogenous reference genes GAPDH and HPRT, and expressed as n-fold levels relative to untreated samples.

Real-time RT-PCR reactions were performed in triplicate and the coefficient of variation (CV), calculated from the three Ct values, was always <1.5%. Reproducibility of the relative mRNA expression was calculated from the results of two experiments in which the procedure was carried out on different retrotranscription products derived from the same mRNA sample. CV was always <10%.

### Determination of PSA Levels

A PSA ELISA kit supplied by Abnova (Taiwan Corporation, Taiwan) was used according to the manufacturer’s instructions. The concentration of PSA was measured spectrophotometrically at 450 nm in culture medium.

### BrdU Incorporation

After *in vivo* labeling with 100 µM BrdU (Sigma), quiescent cells on coverslips were fixed and permeabilized. BrdU incorporation was analyzed by immunofluorescence using diluted (1∶50 in PBS) mouse monoclonal anti-BrdU antibody (clone BU-1, from GE Healthcare), as previously reported [35]. Mouse antibody was detected using diluted (1∶200 in PBS) Texas red-conjugated goat anti-mouse antibody (Jackson Laboratories).

### Immunofluorescence Analysis

Cells on coverslips were fixed and permeabilized [36]. Wild-type hAR ectopically expressed in Cos-7 cells was visualized [37] using the rabbit polyclonal anti-C19 antibody (Santa Cruz). The primary antibody was detected using diluted (1∶100 in PBS) Texas red-conjugated goat anti-rabbit antibody (Jackson Laboratories). Coverslips were finally stained with Hoechst 33258, inverted and mounted in Mowiol (Calbiochem). Fields were analyzed with a DMBL Leica (Leica Microsystems S.r.l., Milan, Italy) fluorescent microscope using an HCXPL Apo 63× oil objective. Images were captured using DC480 camera (Leica) and acquired using FW4000 (Leica) software, as described [36], [37].

### Lysates and Western Blot Analysis

Cell lysates (at 2 mg/ml protein concentration) were prepared as previously described [32]. Cyclin D1, p27 and CDK4 were detected using the appropriate antibodies [38]. AR was detected, using the rabbit polyclonal anti-AR antibodies (C-19; Santa Cruz), as reported [36] Immune-reactive proteins were revealed using the ECL detection system (from GE Healthcare).

### *In vivo* Experiments

Five- to six-week old male SCID C.B-17/IcrHanHsd-Prkdcscid mice were purchased from Harlan Laboratories (Correzzana, Italy). Six-to 8-week old CD-1 male nude (nu/nu) mice were purchased from Charles River Laboratories (Calco, Italy). All the animal experiments were performed at the Animal Facility (SAFU) of Regina Elena National Cancer Institute in Rome, Italy. At the time in which the experiments were performed, there was no active Ethical Committee for Animal Research at Regina Elena National Cancer Institute. However, the Animal Facility at the Institute had received full authorization to perform in vivo experiments from the Italian Ministry of Health, which also approved the present study. All procedures involving animals and their care were conducted in conformity with institutional guidelines, which are in compliance with national (D.L. No. 116, G.U., Suppl. 40, Feb. 213 18, 1992; Circolare No. 8, G.U., July 1994) and international laws (EEC Council Directive 86/609, OJ L 358. 1, Dec 12, 1987; Guide for the Care and Use of Laboratory Animals, United States National Research Council, 1996). The animals were euthanized for ethical reasons by cervical dislocation when tumors reached a mean of 3.0 g in weight or when they became moribund during the observation period.

For (*R*)-**9** toxicological experiments, healthy mice were treated orally by daily gavage with 10, 25, 50 and 100 mg/kg of (*R*)-bicalutamide or (*R*)-**9** for 28 consecutive days. For antitumor activity experiments, mice were injected subcutaneously in the flank with VCaP, LNCaP and LNCaP-RBic prostate cancer cells at 10^6^ cells/mouse in 100 µl of solution composed of 50% Matrigel and 50% serum-free medium. After about 1 month (when a tumor mass of 5–150 mg was evident) mice were randomized, divided in groups and treatment was started. Mice were treated orally by daily gavage for.

4 consecutive weeks with (*R*)-bicalutamide, (*R*)-**9** or Casodex® at 10 mg/Kg. Compounds were dissolved in 80% PEG-400 and 20% Tween-80. Control mice were treated with vehicle for the same treatment period. Five mice for each group were evaluated. Tumor size was measured three times a week in two dimensions by a caliper and tumor weight was calculated using the following formula: a×b^2^/2, where a and b are the long and short diameter of the tumor, respectively.

Antitumor efficacy of treatments was assessed by the following end points: %TWI, percent tumor weight inhibition; (b) tumor growth delay, evaluated as T−C, where T and C are the median times for treated and control tumors, respectively, to achieve equivalent size (*i.e.* 1000 mg); c) stabilization, regression or complete response evinced by palpability.

### Ethics Statement

All procedures involving animals and their care were conducted in conformity with institutional guidelines, which are in compliance with national (D.L. No. 116, G.U., Suppl. 40, Feb. 213 18, 1992; Circolare No. 8, G.U., July 1994) and international laws (EEC Council Directive 86/609, OJ L 358. 1, Dec 12, 1987; Guide for the Care and Use of Laboratory Animals, United States National Research Council, 1996).

### Statistical Analysis

For the analysis of PSA protein assay, differences among values observed after the various treatments were analysed using the Student’s t-test for unpaired observations.

A *P* value <0.05 was considered significant. For the analysis of quantitative real-time PCR experiments, one-way ANOVA with Dunnett’s post test was carried out using GraphPad Prism version 4.00 for Windows (GraphPad Software, San Diego California USA). Data obtained from BrdU incorporation, ARE-luc reporter gene and nuclear translocation assays were analyzed by paired *t-test*. A *P* value <0.05 was considered significant. For *in vivo* experiments, the Student’s t-test (unpaired, two-tailed) was used to compare mean values (Software Primer of Biostatistics, McGraw-Hill, New York, NY, USA). Differences were considered statistically significant when *P*<0.05.

## Results

### Compound Structure

**Figure 1:**
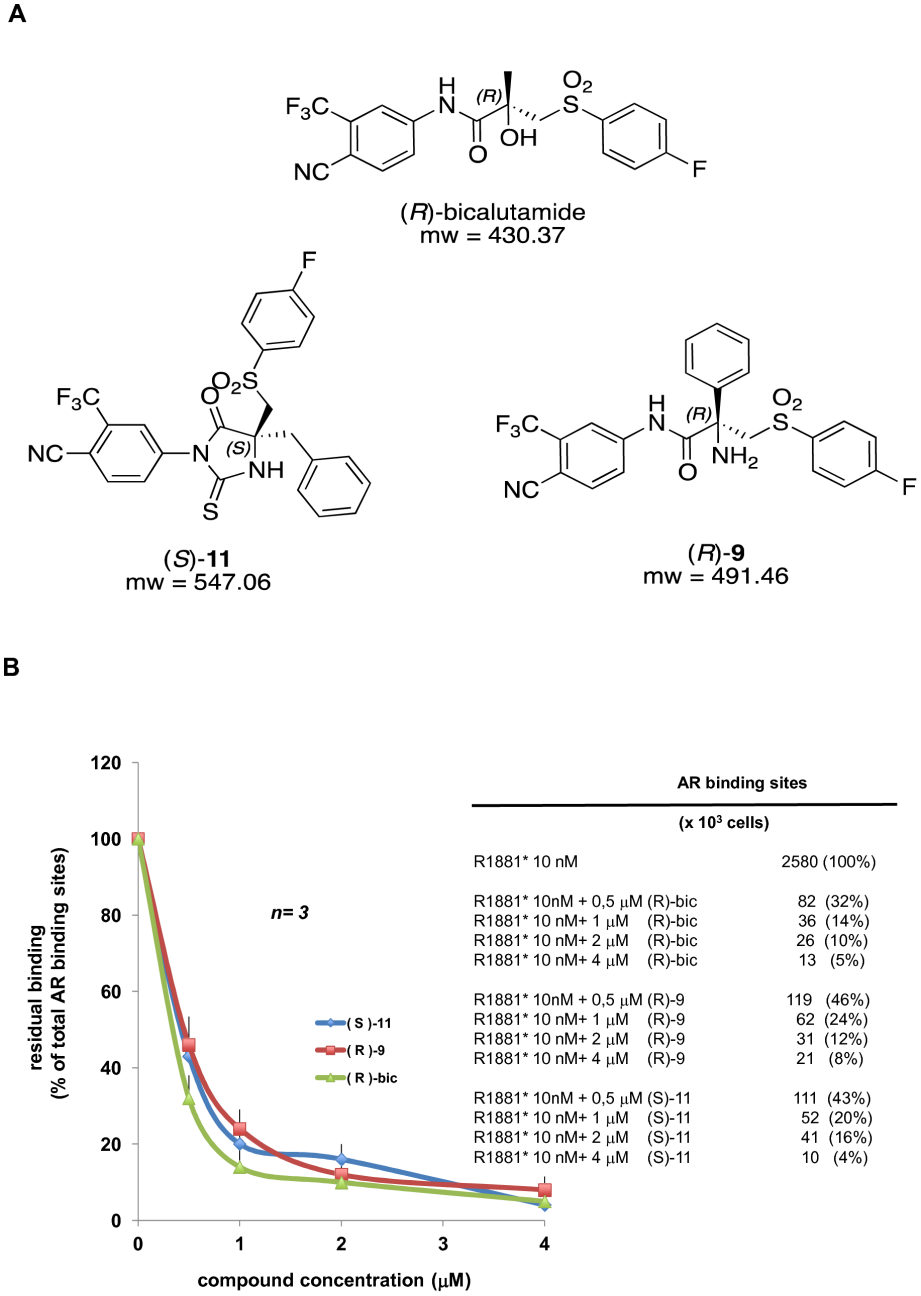
Structure compounds and their AR-specific binding properties. (**A**) Chemical structure and molecular weight (m.w.) of (*R*)-bicalutamide, (*S*)-11 and (*R*)-9. (**B**) AR ligand binding displacement analysis in LNCaP cells**.** Quiescent LNCaP cells were incubated with 10 nM [3H] R1881 in the absence or presence of the indicated excess (from 0.5 µM to 4 µM) of radio inert compounds. Intracellular radioactivity was assayed. Inset in panel B shows the AR binding sites assayed in 10^3^ cells incubated with 10 nM of [3H] R1881 (R1881*) in the absence or presence of the indicated excess of unlabeled (*R*)-**9**, (*S*)-**11** or (*R*)-bicalutamide (*R*)-bic. Data from three different experiments were collected and residual binding was calculated and expressed as % of total AR binding sites. *n* =  number of experiments. The statistical significance of results was also evaluated by the paired *t* test and P values <0.005 were considered significant. No significance was attributed to the difference in the residual binding between the cells incubated with 10 nM [3H] R1881 in the presence of (*S*)-11 or (*R*)-9 and those incubated with 10 nM [3H] R1881 in the presence of (*R*)-bicalutamide ((*R*)-bic). LNCaP cells were also incubated with 10 nM [3H] R1881 in the absence or presence of 100-fold excess (1 µM) of unlabeled R1881 or Casodex®. Residual binding was 13% and 14% for unlabeled R1881 or Casodex®, respectively.

Our work focused on the identification of potential lead compounds of a new class of selective androgen receptor modulators (SARMs), synthesized by an innovative and highly diastereoselective methodology, for further preclinical and clinical development. The antagonistic and agonistic activities of about 30 different nonsteroidal, synthetic AR ligands through a structure-activity relationship study have already been described [28]. In particular, we assessed the *in vitro* antitumor activity of two novel bicalutamide-like propanamide molecules, (*S*)-**11** and (*R*)-**9**, (Fig. 1A). Their chemical structures differ from that of bicalutamide in the presence of a nitrogen atom instead of the hydroxyl group at the central carbon atom, and in the substituent at the chiral stereocenter, *e.g*. a benzyl and a phenyl group, respectively. Moreover, (*S*)-**11** is characterized by the presence of a hydantoin moiety, which further influences its steric hindrance.

### Binding Displacement Studies

We first evaluated the ability of (*S*)-**11** and (*R*)-**9** to specifically displace the ligand binding activity of AR in whole LNCaP cells. To this end, quiescent cells were incubated with 10 nM of [3H] R1881 in the absence or presence of the indicated excess of radio-inert compounds. The cells were made quiescent by treating serum with dextran-charcoal to remove free steroids and then collected by gently scraping to preserve the binding of membrane AR in order to consider it as part of the binding results. Results from different independent experiments were analyzed, revealing that both (*S*)-**11** and (*R*)-**9** compounds displaced the [3H] R1881 binding by about 45% and 80% when used at 0.5 µM and 1 µM, respectively (Fig. 1B and inset). Almost total [3H] R1881 displacement was detected when each compound was used at 2 µM or 4 µM (Fig. 1B and inset). Similar data were observed by using unlabeled R1881 or Casodex® (Fig. 1B legend) or when binding displacement analysis was performed in Cos cells ectopically-expressing hAR (data not shown). The anti-androgen (*R*)-bicalutamide substantially behaved like (*S*)-**11** and (*R*)-**9**, although it was slightly more effective than (*S*)-**11** and (*R*)-**9** in displacing the AR ligand binding when used at low concentrations (0.5 or 1 µM). From a statistical point of view (Fig. 1B legend), these differences appear negligible. Overall, these findings indicate that (*S*)-**11** and (*R*)-**9** compounds bind AR.

### Cytotoxic Activity in vitro

**Figure 2:**
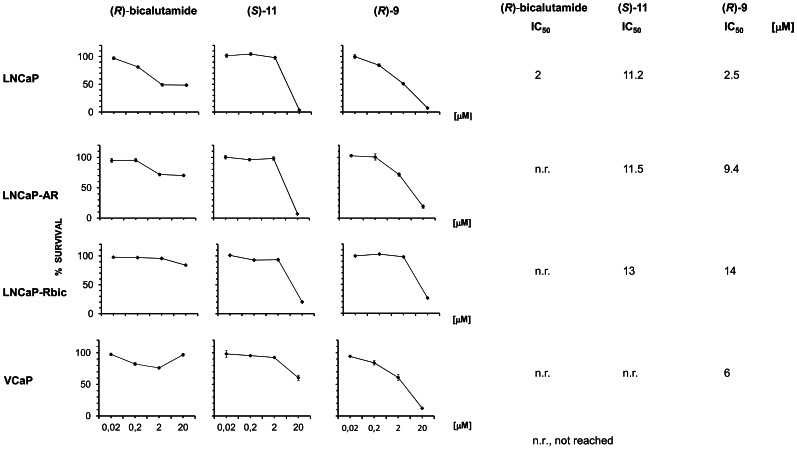
Cytotoxic activity *in vitro*. Cytotoxic activity of (*R*)-bicalutamide, (*S*)-**11**and (*R*)-**9** in human prostate cancer cell lines LNCaP, LNCaP-Rbic, LNCaP-AR and VCaP after a 144-hour exposure, measured by SRB assay (average of three independent experiments). The concentrations (µM) of (*R*)-bicalutamide, (*S*)-**11** and (*R*)-**9** causing 50% decrease in cell survival (IC_50_) are shown to the right of the curves.

We then assessed the *in vitro* cytotoxic activity of (*R*)-**9** and (*S*)-**11** in a panel of prostate cancer cell lines at different stages of hormone responsiveness and representative of different pathological features of human prostate cancer (Fig. 2).

Cells were exposed to scalar drug concentrations ranging from 0.02 µM to 20 µM for 144 hours. (*R*)-bicalutamide showed no dose-effect curves in the resistant LNCaP-Rbic cell line or in VCaP cells harboring high levels of wild type AR. Although a weak dose-effect trend was observed in the cell lines naturally expressing AR (LNCaP) or cells engineered to overexpress the receptor (LNCaP-AR), IC_50_ (2 µM) was only observed in LNCaP cell line (Fig. 2).

(*S*)-**11** showed a modest activity at all but the highest concentration (20 µM) at which we observed a modest cytotoxic effect in VCAP cells (Fig. 2). In the other cell lines a strong cytocidal effect was detected with IC_50_ values ranging from 11.2 µM to 13 µM. (*R*)-**9** produced an effect similar to that of (*S*)-**11** in only LNCaP-Rbic but induced a dose-related and strong cytotoxic effect in the other cell lines, naturally or artificially expressing AR, always reaching IC_50_ values, even in VCaP cells (6 µM).

### Influence on Hormonal Stimulus

**Figure 3:**
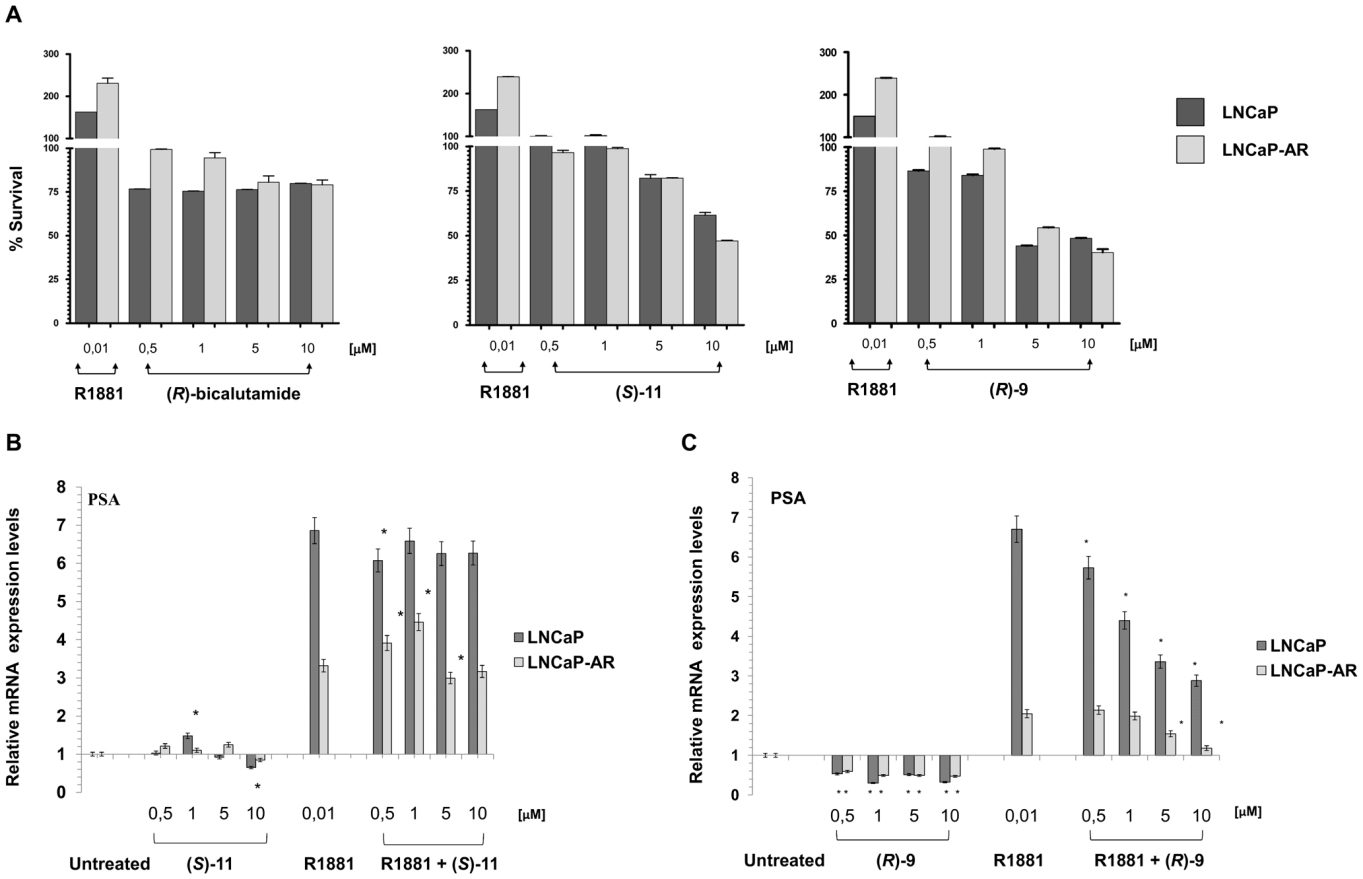
Interference of the anti-androgens on the effect of R1881 in naïve LNCaP and derivative LNCaP-AR lines. (**A**) Evaluation by SRB assay of the antitumor acitivity of scalar concentrations of (*R*)-bicalutamide, (*S*)-**11** or (*R*)-**9** in hormone-responsive LNCaP and AR-overexpressing LNCaP-AR cells, in the presence or not of the synthetic androgen, R1881 (10 nM). Bars represent the mean of two independent experiments**.** (**B**)**,** (**C**) Evaluation of mRNA levels of PSA gene after a 24-hour exposure to different concentration of (*S*)-**11** (C) or (*R*)-***9*** (D) in the presence or absence of R1881 (points are mean of two independent experiments, *P>0.05).

We also investigated the interference of the antiandrogens on the effect of R1881 in naïve LNCaP and derivative LNCaP-AR lines, the latter engineered to express higher levels of wild-type AR ((Fig. 3A). Prolonged exposure to R1881 10 nM increased cell proliferation of the prostate cancer lines by about 1.5–2.5-fold. The concomitant exposure of cells to R1881 and (*R*)-bicalutamide, (*S*)-**11** or (*R*)-**9** for 72 hours suppressed the growth stimulus given by the synthetic androgen. In particular, (*R*)-**9** proved to be the most effective in inhibiting the hormonal proliferative stimulation, starting from a 5-µM concentration. Furthermore, as expected, a significant increment (*P*<0.01) in PSA levels was observed in the culture medium of LNCaP (43%) and LNCaP-AR (62%) cells after a 48-hour exposure to 10 nM of the synthetic androgen R1881 (Fig. S1). In contrast, all the antiandrogen compounds used at concentrations of 20 µM inhibited PSA secretion in the culture medium of both cell lines. In particular, both (*R*)-**9** and (*S*)-**11** caused a reduction in PSA levels significantly higher (*P*<0.01) than that produced by (*R*)-bicalutamide (*P*<0.01).

We also investigated the early modulation of AR transcriptional activity exerted by the two new molecules at exposure times and concentrations not capable of inducing massive cell killing.

A significant increment in PSA mRNA levels was observed (*P*<0.01) in both cell lines after a 24-hour exposure to 10 nM R1881 (Fig. 3 B-C). A 24-hour exposure to (*S*)-**11**, alone or in combination with R1881, induced a modest reduction in PSA mRNA levels in both cell lines. Conversely, a 24-hour exposure to (*R*)-**9** alone induced a significant reduction of PSA mRNA expression in LNCaP and LNCaP-AR starting from lowest concentration used (0.5 µM). When a 24-hour exposure of R1881 preceded that of (*R*)-**9**, a significant reduction in PSA mRNA expression with respect to that exhibited by cells exposed to R1881 alone was observed starting from 0.5 µM in LNCaP and from 5 µM in LNCaP-AR, respectively (*P*<0.005). The data obtained seem to indicate that (*R*)-**9** at low concentrations is more effective than (*S*)-**11** in inhibiting the transcriptional activity of AR.

### Effect on BrdU Incorporation

**Figure 4:**
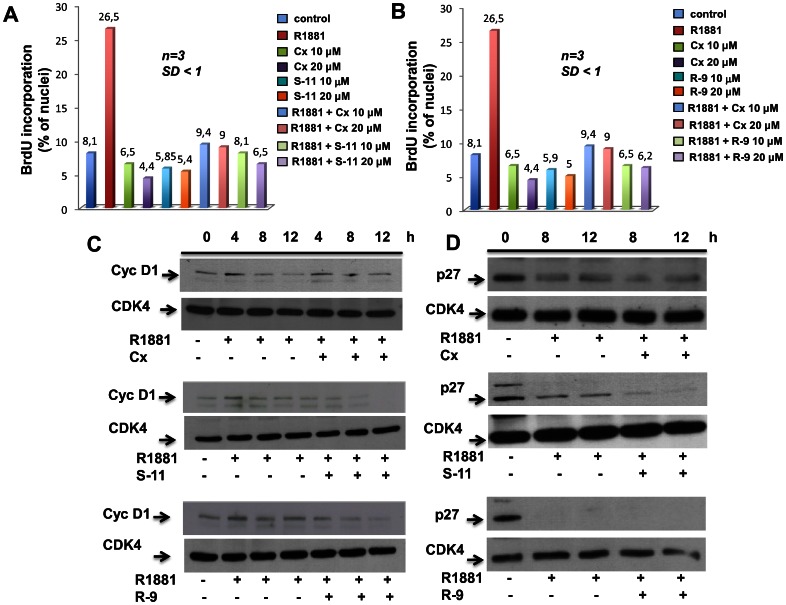
(*S*)-11- and (*R*)-9-induced inhibition of G1/S progression in androgen-treated LNCaP cells. In **A and B**, quiescent LNCaP cells on coverslips were left untreated (control) or treated for 18 hours with the synthetic androgen R1881 (10 nM) in the absence or presence of the indicated antagonists (used at 10 or 20 µM). After *in vivo* pulsing with 100 µM BrdU, BrdU incorporation was analyzed by immunofluorescence and expressed as % of total nuclei. In **A** and **B**, the numbers at the top of each bar represent the mean of three independent experiments (*n = 3*), with standard deviation (SD) <1. The statistical significance of results in **A** and **B** was assessed with the paired *t* test. *P* values were <0.005 for cells stimulated with 10 nM R1881. No significance was attributed to the difference in BrdU incorporation between control cells and cells stimulated with 10 nM R1881 in the presence of Casodex®, (*S*)-**11** (**A**) or (*R*)-**9** (**B**). In **C** and **D,** quiescent LNCaP cells were left untreated or were treated for the indicated times with 10 nM R1881, in the absence or presence of 10 µM of the indicated antagonists (Casodex®, Cx; (*S*)-**11** or (*R*)-**9**). Lysate proteins were analyzed by Western blot using antibodies directed against cyclin D1 (Cyc D1) in **C,** or p27 in **D**. Filters were stripped and re-probed using the rabbit polyclonal anti CDK-4 antibody as a loading control. Western blots in **C** and **D** are representative of two different experiments. In **C**, a 3.3-fold increase in the levels of 4-hr hormone-induced Cyc D1 expression was detected using the NIH ImageJ program. Casodex, (*S*)-**11** and (*R*)-**9** inhibited this increase (16% 80% and 66% for Casodex®, for S-11 and R-9, respectively) to varying degrees.

We evaluated the effect of both compounds on the androgen-induced DNA synthesis of LNCaP cells in three different experiments. (*R*)-**9** (Fig. 4A) and (*S*)-**11** (Fig. 4B) significantly inhibited BrdU incorporation stimulated by 10 nM R1881 treatment of LNCaP cells made quiescent using phenol red-free medium and dextran charcoal-treated serum. The inhibitory effect was similar or even stronger than that obtained using the same concentration range (10–20 µM) of Casodex (Fig. 4 A and B) or (*R*)-bicalutamide (Table 1). (*R*)-**9** and (*S*)-**11** once again significantly inhibited BrdU incorporation stimulated by 10 nM R1881 treatment of LNCaP-AR cells made quiescent as described above for LNCaP (Fig. S2).

**Table 1:** Effect of (R)-bicalutamide, (*R*)-9 and (*S*)-11 on BrdU incorporation induced by R1881 in LNCaP cells.

BrdU (% of total nuclei)	
Control	8.6
R1881	30
(*R)-*bic 10 µM	6.8
(*R)-*bic 20 µM	6.3
(*S*)-**11** 10 µM	5.9
(*S*)-**11** 20 µM	5.8
(*R*)-**9** 10 µM	6.7
(*R*)-**9** 20 µM	5
R1881+ (*R)-*bic 10 µM	9
R1881+ (*R)-*bic 20 µM	9.3
R1881+ (*S*)-**11** 10 µM	8.4
R1881+ (*S*)-**11** 20 µM	6.5
R1881+ (*R*)-**9** 10 µM	6.9
R1881+ (*R*)-**9** 20 µM	6.5

Quiescent LNCaP cells on coverslips were left untreated (control) or treated for 18 hours with the synthetic androgen R1881 (10 nM), in the absence or presence of the indicated antagonists (used at 10 µM or 20 µM). After *in vivo* pulsing with 100 µM BrdU, BrdU incorporation was analyzed by immunofluorescence and expressed as % of total nuclei. The numbers represent the mean of three independent experiments, with standard deviation <1.4. The statistical significance of results was also evaluated by the paired *t* test. No significance was attributed to the difference in BrdU incorporation between the control cells and cells stimulated with 10 nM R1881 in the presence of (*R)*-bicalutamide (*R*)-bic or (*S*)-**11** or (*R*)-**9**.

### Modulation of G1/S Progression

We next analyzed the effect of (*R*)-**9** and (*S*)-**11** on cyclin D1 and p27 expression in LNCaP cells. Both of these proteins are, indeed, modulated by androgens [39]. The Western blot in Fig. 4C shows an increase of cyclin D1 levels after 4 hours and a slight decrease after prolonged androgen treatment (8 and 12 hours). The activity of the two inhibitors on cyclin D1 expression was compared with that of Casodex®. In agreement with previous results [40], the upper panel in C showed that Casodex did not significantly affect cyclin D1 expression levels. A substantial reduction in cyclin D1 expression was observed by the addition of **(** *S* **)-11** (middle panel) or (*R*)-**9** (lower panel). The androgen R1881 also induced p27 downregulation (Fig. 4D), which was unaffected by any of the three antagonists (Casodex®, (*R*)-**9** or (*S*)-**11**) in LNCaP cells.

### Interference in AR-mediated Transcriptional Activation

We then investigated the ability of the two compounds, (*R*)-**9** and (*S*)-**11,** to interfere with androgen-stimulated transcriptional activation in LNCaP cells. Fig. S3 shows that (*R*)-**9** (A) and (*S*)-**11** (B) significantly inhibited the androgen-triggered ARE-luc activity in our reporter gene assay. Similar findings were observed in LNCaP cells treated with *(R)-*bicalutamide or Casodex® (Fig. S3) or in Cos-7 cells ectopically expressing hAR (Fig. S4).

### Modulation of AR Nuclear Translocation

Neither (*S*)-**11** nor (*R*)-**9** affected AR nuclear translocation induced by a 60-minute hormonal stimulation in Cos-7 cells ectopically expressing hAR. Similar findings were observed using the antiandrogen Casodex® (Panel A, Fig. S5). The images (B and C)are representative of one experiment in panel A. Of note, (*S*)-**11**, in contrast with Casodex® and (*R*)-**9**, induced a significant AR nuclear translocation when used alone in our assay.

### Mouse Xenograft Studies

**Figure 5:**
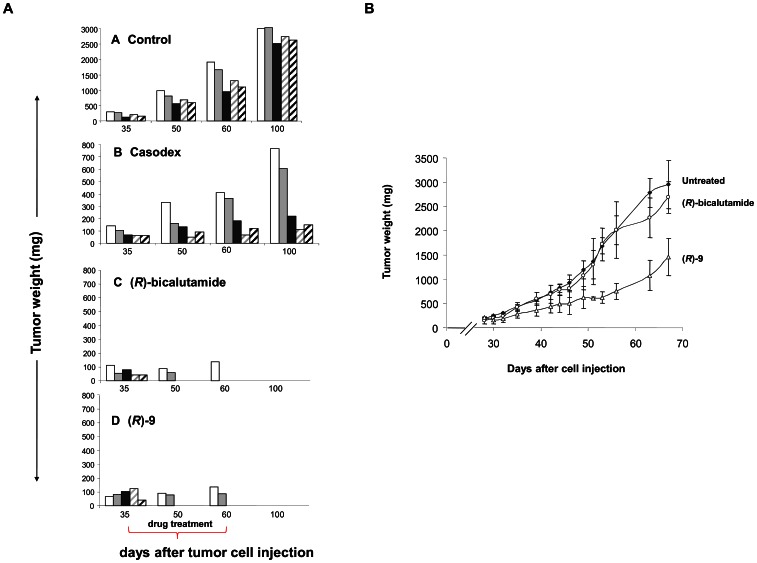
Mouse xenograft studies. (**A**) (*R*)-**9** and (*R*)-bicalutamide have high therapeutic efficacy against VCaP xenografts. CD1 male nude (nu/nu) mice were injected subcutaneously with 1×10^6^ VCaP cells/mouse and treatment was started 35 days after tumor cell injection, when a tumor mass of 50–150 mg was evident. Mice were treated orally by daily gavage for 4 consecutive weeks with Casodex® (B), (*R*)-bicalutamide (C) or (*R*)-**9** (D) at 10 mg/Kg. Control group (A) received vehicle for the same period of treatment. Five mice per group were evaluated: 1^st^ mouse (white bar), 2^nd^ mouse (light grey bar), 3^rd^ mouse (dark grey bar), 4^th^ mouse (grey striped bar) and 5^th^ mouse (black striped bar). (**B**) (*R*)-**9** treatment inhibited the growth of *(R)-*bicalutamide-resistant LNCaP tumors. SCID mice were injected subcutaneously with 1×10^6^ LNCaP-RBic cells/mouse and treatment started on 28^th^ day after tumor cell injection. Mice were given 10 mg/Kg of *(R)-*bicalutamide or (*R*)-**9** *per os* for four consecutive weeks. Control group received vehicle for the same treatment period. Groups: (♦), control; (o), *(R)-*bicalutamide; (▵), (*R*)-**9**. Points are means with SD (bars).

We subsequently evaluated the therapeutic efficacy of the new antiandrogen (*R*)-**9** in mice implanted with the different prostate cancer cell lines. The treatment schedule was chosen on the basis of preliminary experiments performed on healthy mice in which antiandrogen treatment was well tolerated (doses of 10–100 mg/kg given by oral gavage for 28 consecutive days (data not shown). On the 35th day after cell injection the mice were treated orally for 4 consecutive weeks with Casodex®, (*R*)-bicalutamide or (*R*)-**9** at 10 mg/Kg. After the end of the treatment the mice were maintained under observation (until the 100^th^ day after tumor cell injection) in order to continue tumor growth evaluation. In all of the mice treated with Casodex® we observed only a delay in tumor growth with respect to control mice. In contrast**,** (*R*)-**9** compound elicited an impressive antitumor effect on VCaP xenografts (Fig. 5A). In fact, while control mice treated with vehicle alone showed a progression in tumor growth, two out of five mice treated with (*R*)-**9** had obtained disease stabilization on the 49^th^ day after tumor cell implant (about two weeks’ treatment) and the remaining three showed a complete response. The efficacy of (*R*)-**9** further increased, a complete response registered in all the animals treated 98 days after tumor cell implant. These animals showed no signs of relapse and were considered cured after being administered the new antiandrogen (*R*)-**9**.

Similar results to those obtained with (*R*)-**9** were observed with (*R*)-bicalutamide, thus confirming the efficacy of androgen ablation in this prostate cancer line expressing wild-type AR.

The good antitumor efficacy of (*R*)-**9** was also observed in LNCaP xenografts. Such treatment produced 66% tumor weight inhibition (TWI) and a 13-day tumor growth delay compared to 50% and 11 days, respectively, observed after *(R)-*bicalutamide administration (Table S1). Interestingly, (*R*)-**9** treatment also maintained its antitumor efficacy against (*R*)-bicalutamide resistant LNCaP tumors. In fact, while.

(*R*)-bicalutamide treatment did not promote substantial growth inhibition in LNCaP-Rbic, (*R*)-**9** induced 56% TWI and tumor growth delay of 16 days, both values significantly different from those obtained in (*R*)-bicalutamide-treated mice (*P* = 0.033 and 0.044, respectively) and control mice (*P* = 0.014 and *P* = 0.017, respectively) (Fig. 5B, Table S1). Finally, it is important to outline that treatment with (*R*)-**9** was well tolerated as no toxic deaths or body weight loss were observed during or after the end of treatment.

## Discussion

Clinical and PSA response to second-line hormone therapies currently used in castration-resistant prostate cancer (CRPC) and based on the use of antiandrogens alone or in combination with corticosteroids varies from 20% to 40%, with a median duration of about 5 months and very few durable (2–4 years) responses [41], [42]. The limited efficacy of second-line treatment is probably the result of incomplete AR inhibition.

Great interest has recently been shown in two new compounds, MDV3100, an antiandrogen specifically designed for use in AR-overexpressing prostate cancer [43], and abiraterone acetate, a CYP17A inhibitor that blocks steroid biosynthesis in the adrenal gland and possibly within the tumor [44]. However, preliminary results from ongoing phase III trials reveal that not all patients respond to MDV3100 or abiraterone treatment and that resistance often develops in initial responders. In both cases, as with other prostate cancer treatments, disease progression most frequently correlates with a rise in PSA levels, indicating reactivation of the androgen receptor [45].

Our work focused on the identification of potential lead compounds of a new class of SARMs, synthesized by an innovative, highly diastereoselective synthetic methodology, [28] for further preclinical and clinical development. First, we tested the activity of the two novel compounds on LNCaP cells, which are sensitive to the growth inhibitory effect of Casodex®, despite harboring a mutation at codon 877 of the AR ligand-binding domain. This mutation alters AR ligand specificity leading to receptor activation by progesterone, estradiol, cyproterone acetate, nilutamide and hydroxylflutamide [46]. A long exposure to both novel compounds exerted an impressive antitumoral effect. Their activity was compared in *in vitro* experiments with that of (*R*)-bicalutamide, the active enantiomer of the most widely prescribed non steroidal antiandrogen, Casodex®, which showed a lower cytotoxic effect than that exerted by the two novel molecules.

We also tested the *in vitro* activity of both compounds on LNCaP-(R)-bic, a subclone derived and isolated in our laboratory from LNCaP cell line and made resistant to *(R)-*bicalutamide, thus representative of clinical hormone-refractory or castration-resistant prostate cancer. Both (*R*)-**9** and (*S*)-**11** maintained a high cytotoxic activity, indicating their potential usefulness in this frequent clinical condition where treatments based on conventional chemotherapeutic drugs have limited efficacy.

Finally, we tested the compounds on experimental models expressing high levels of AR and representative of clinical AR gene amplification reported in 25%–30% of patients with CRPC. Such a condition is present at very low rates (1–2%) in patients with primary prostate cancer, indicating that AR amplification is involved in the development of CRPC [47]–[49]. In particular, cytotoxicity was maintained by (*R*)-**9** in this hormone-resistant condition in either cell line naturally or artificially [43] overexpressing wild type AR, giving proof of the high binding affinity of this molecule for AR. This was further confirmed by the displacement experiments performed in the presence of 10 nM radio-labeled R1881 (Fig. 1B). Such a concentration has also been used in both binding affinity and displacement studies [33], [50], [51].

Unlike Casodex®, (*S*)-**11** and (*R*)-**9** inhibited DNA synthesis by blocking androgen-induced cyclin D1 expression to varying degrees in LNCaP cells. As cyclin D1 deregulation is a hallmark of prostate cancer [52], the use of these two inhibitors can be envisaged in prostate tumors that exhibit altered cyclin D1 expression.

The androgen-regulated protein, PSA, is currently used to monitor recurrence in patients with advanced prostate cancer. The substantial reduction in PSA protein levels in the culture medium and in PSA mRNA expression in both cell lines after exposure to either drug, even in the presence of the androgen, suggests that they both prevent AR binding with agonist molecules, thus inhibiting AR-mediated transcriptional activity. Reporter gene assays performed in LNCaP and Cos-7 cells further support this conclusion. Regardless of the cell system, (*R*)-**9** and (*S*)-**11** efficiently inhibited androgen-induced gene transcription without significantly affecting the nuclear translocation of AR. These latter findings call for some comment. In contrast to (*R*)-**9** and Casodex®, (*S*)-**11** compound significantly enhanced AR nuclear translocation when used alone in our assay. Although such results support the hypothesis that (*S*)-**11** acts as a partial agonist, the fact that it does not activate AR-mediated gene transcription or DNA synthesis when used alone (Fig. 4A and C; Fig. S2, S3 and S4 definitively confutes it. (*S*)-**11,** on the other hand, may facilitate AR nuclear import by inducing AR conformational changes that either unmask nuclear import signals (NLSs) or mask nuclear export signals (NESs) of AR.

As (*R*)-**9** almost always showed superior activity to that of (*S*)-**11** in *in vitro* cytotoxicity experiments**,** we chose the former for animal experiments. Preliminary *in vivo* toxicity experiments carried out on healthy SCID mice with compound (*R*)-**9** showed generally good tolerance of the drug, highlighting its potentially positive safety profile. Furthermore, in *in vivo* antitumor efficacy experiments, (*R*)-**9** confirmed its effectiveness against castration-resistant prostate cancer models. In fact, the compound induced complete responses in VCaP xenografts similarly to the (*R*)-bicalutamide, the active enantiomer, not currently used in clinical practice, of the most widely prescribed antiandrogen Casodex®, the latter showing modest antitumor activity in the same experimental model. About this, the data obtained in VCaP xenografts in part differs from that observed *in vitro* experiments, where (*R*)-bicalutamide showed a modest cytotoxic activity and always lesser than that exhibited by (*R*)-**9.** This apparent discrepancy may be due to the good pharmacokinetic profile of the Casodex® active enantiomer and its consequent high bioavailability in *in vivo* models.

Moreover, in the experiments conducted on the *in vitro* and *in* *vivo* models representative of recurrent disease, (*R*)-**9** strongly inhibited tumor growth, highlighting the potential efficacy of the compound in this common clinical condition.

In conclusion, the significant *in vitro* and *in vivo* antitumor activity shown by (*R*)-**9** in advanced prostate cancer models highlights its potential usefulness in advanced human disease where current therapeutic options remain unsatisfactory. The pharmacokinetic profile of (*R*)-**9** is under investigation in our laboratory to improve its bioavailability whilst maintaining its antiandrogenic efficacy.

## Supporting Information

Figure S1 **Secreted PSA level in cell culture media.** Determination of PSA levels in culture medium of LNCaP and LNCaP-AR cells after exposure to R1881, (*R*)-bicalutamide, (*S*)-**11** and (*R*)-***9*** (mean ± s.d. of three independent experiments; *P<0.01).
(TIF)

Figure S2 **Effect on BrdU incorporation in LNCaP-AR cells.** Quiescent cells on coverslips were used in **A** and **B**. Cells were either left untreated (control) or were treated for 18 hours with the synthetic androgen R1881 (10 nM), in the absence or presence of the indicated antagonists (used at 10 µM or 20 µM). After *in vivo* pulsing with 100μµM BrdU, BrdU incorporation was analyzed by immunofluorescence and expressed as % of nuclei. Mean and standard error of the mean (SEM) are shown in **A** and **B**. *n* represents the number of experiments. The statistical significance of results in **A** and **B** were also evaluated by the paired *t* test. *P* values were <0.005 for cells stimulated with 10 nM R1881. No significance was attributed to the difference in BrdU incorporation between control cells and cells stimulated with 10 nM R1881 in the presence of (*R*)-bicalutamide ((*R*)-bic), (*S*)-**11** (**A**) or (*R*)-**9** (**B**).
(TIFF)

Figure S3 **Interference in AR-mediated transcription in LNCaP cells.** In **A** and **B**, LNCaP cells were transfected with ARE-luc 3416 reporter gene and then made quiescent, as described in the Methods section. Twenty-four hours later, the cells were left untreated (control) or treated for 24 hours with the synthetic androgen R1881 (10 nM) in the absence or presence of the indicated antagonists (used at 10 µM or 20 µM). Luciferase activity was assayed, normalized using β-galactosidase (β -gal) as internal control and expressed as -fold induction. Data from several independent experiments were analyzed. Means and SEMs are shown; *n* represents the number of experiments. The statistical significance of results in **A** and **B** was also evaluated by the paired *t* test. In both panels, *P* values were <0.001 for cells stimulated with 10 nM R1881. No significance was attributed to the difference in ARE-luc induction between control cells and cells stimulated with 10 nM R1881 in the presence of bicalutamide (R-bic), (*S*)-**11** (A) or (*R*)-**9** (**B**). Once again, no significance was attributed to the difference in ARE-luc induction between control cells and cells stimulated with 10 nM R1881 in the presence of Casodex® (Cx ), (*S*)-**11** (A) or (*R*)-**9** (**B**).
(TIFF)

Figure S4 **Interference in AR-mediated transcription in Cos-7 cells ectopically expressing hAR.** In **A** and **B**, AR-negative Cos-7 cells were transfected with pSG5-hAR encoding plasmid together with ARE-luc 3416 plasmid. Control cells were transfected with pSG5 alone. Cells were made quiescent and after 18 hours they were left untreated (control) or treated for 24 hours with the synthetic androgen R1881 (10 nM) in the absence or presence of the indicated antagonists (used at 10 µM or 20 µM). Luciferase activity was assayed, normalized using β-galactosidase (β -gal) as internal control, and expressed as -fold induction. Data from several independent experiments were analyzed. Means and SEMs are shown; *n* represents the number of experiments. In Cos-7 cells ectopically expressing hAR, the difference in ARE-luc induction between untreated cells and those challenged with 10 nM R1881 was significant (*P*<0.005 in **A** and **B**). In the same cells the difference in ARE-luc induction between the cells stimulated with 10 nM R1881 alone and those stimulated with 10 nM R1881 in the presence of bicalutamide (**A** and **B**), (*S*)-**11** (**A**) or (*R*)-**9** (**B**) was also significant (*P*<0.005). In **C**, lysate proteins were analyzed by Western blot using the antibodies directed against the indicated proteins. AR, androgen receptor. The filter was stripped and re-probed with anti-tubulin antibody (tub) as loading control.
(TIFF)

Figure S5 **Effect of (** ***S*** **)-11 and (** ***R*** **)-9 on nuclear translocation of AR.** In **A**, **B** and **C**, AR-negative Cos-7 cells on coverslips were transfected with pSG5-hAR encoding plasmid and then made quiescent. Eighteen hours later, the cells were left untreated (control) or treated for 1 hour with the synthetic androgen R1881 (10 nM) in the absence or presence of 10 µM of the indicated antagonists (Casodex®, Cx). The cells were then analyzed by immunofluorescence for AR, as described in Methods. In **A**, cells expressing exclusively nuclear AR fluorescence were scored. Results from three different experiments were collected and expressed as % of transfected cells. Data from several independent experiments were analyzed. Means and SEMs are shown; *n* represents the number of experiments. In Cos-7 cells ectopically expressing hAR, the difference in nuclear AR between the untreated cells and those challenged with 10 nM R1881 was significant (*P*<0.001). In the same cells the difference in nuclear AR between the un-stimulated cells (control) and those stimulated with 10 nM (*S*)-**11** was also significant (*P*<0.05). Again, the difference in nuclear AR between the cells challenged with 10 nM R1881 and those stimulated with 10 nM R1881 in the presence of Casodex® or (*S*)-**11** or (*R*)-**9** was not significant. Panels **B** and **C** show representative images from one experiment in A. The arrows indicate the cells showing exclusively nuclear AR. Bar, 10 µm.
(TIFF)

Table S1 Antitumor efficacy of antiandrogen on LNCaP and LNCaP-Rbic xenografts.
(DOCX)
