# Supplementary figures and images for: Effect of Small Molecules Modulating Androgen Receptor (SARMs) in Human Prostate Cancer Models
Source: PLoS One. 2013 May 8;8(5):e62657. doi: 10.1371/journal.pone.0062657 (PMC3648536; doi:10.1371/journal.pone.0062657)

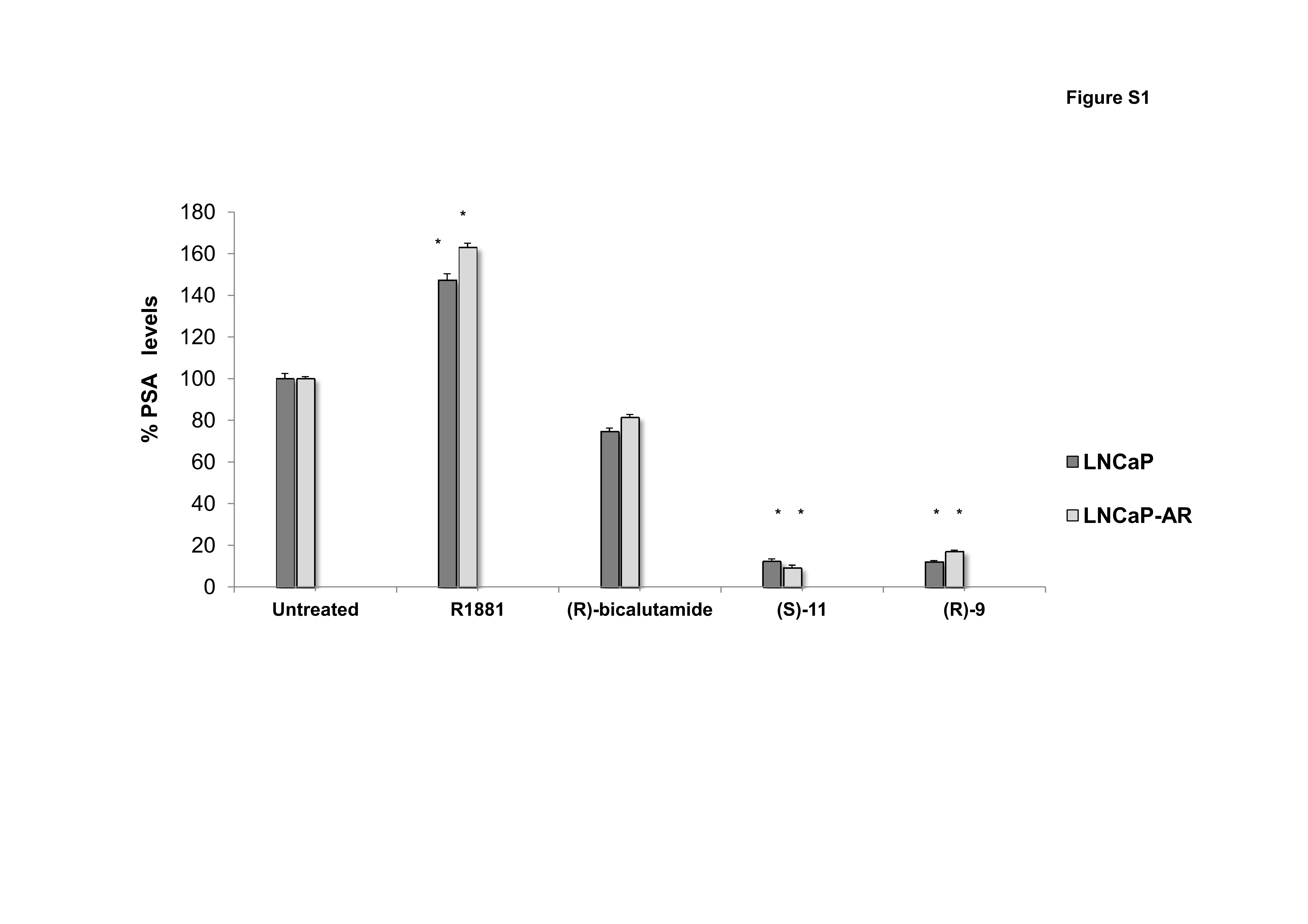

Supplement: Figure S1 — Secreted PSA level in cell culture media. Determination of PSA levels in culture medium of LNCaP and LNCaP-AR cells after exposure to R1881, (R)-bicalutamide, (S)-11 and (R)-9 (mean ± s.d. of three independent experiments; *P<0.01). (TIF) [file pone.0062657.s001.tif]

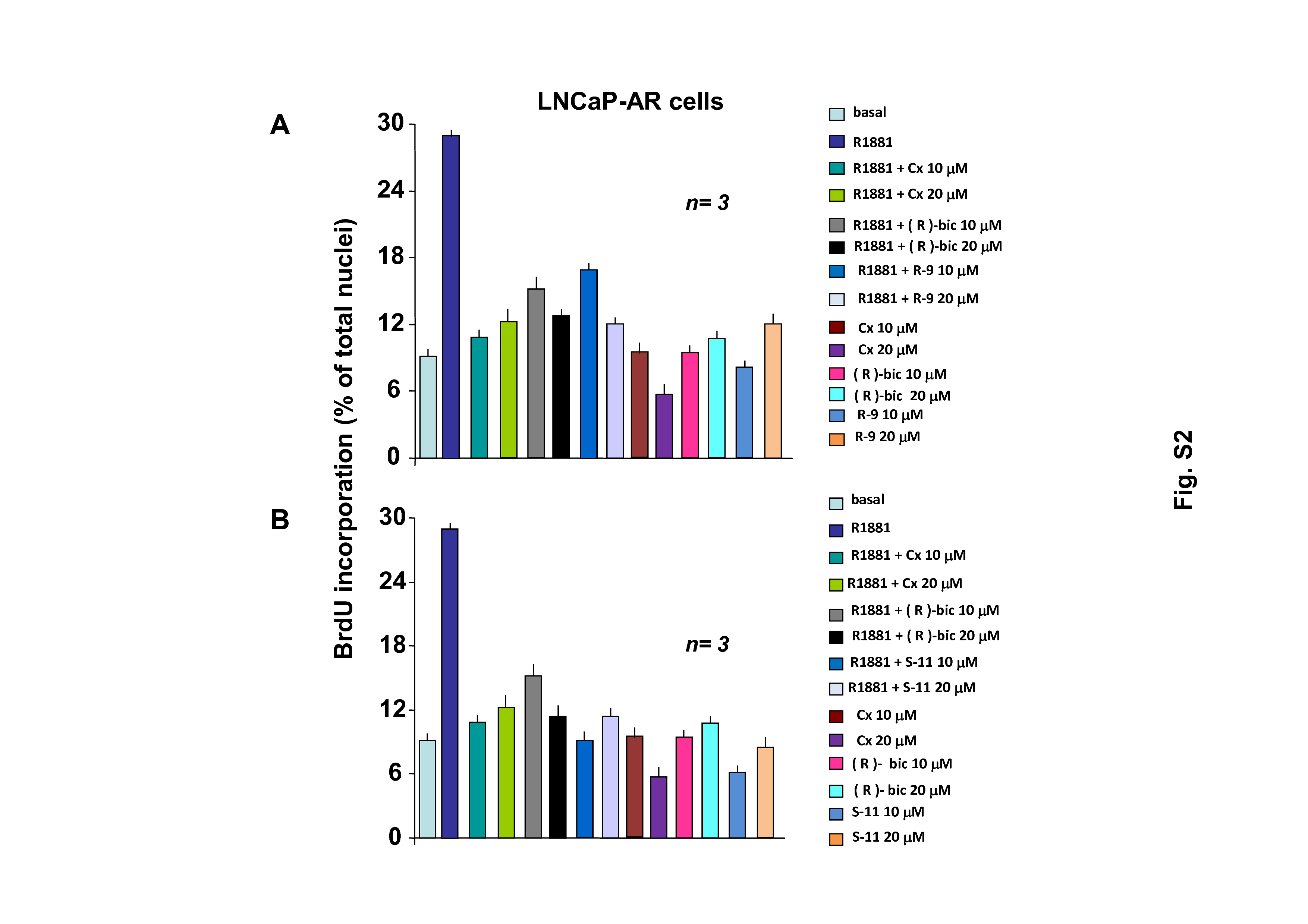

Supplement: Figure S2 — Effect on BrdU incorporation in LNCaP-AR cells. Quiescent cells on coverslips were used in A and B. Cells were either left untreated (control) or were treated for 18 hours with the synthetic androgen R1881 (10 nM), in the absence or presence of the indicated antagonists (used at 10 µM or 20 µM). After in vivo pulsing with 100μµM BrdU, BrdU incorporation was analyzed by immunofluorescence and expressed as % of nuclei. Mean and standard error of the mean (SEM) are shown in A and B. n represents the number of experiments. The statistical significance of results in A and B were also evaluated by the paired t test. P values were <0.005 for cells stimulated with 10 nM R1881. No significance was attributed to the difference in BrdU incorporation between control cells and cells stimulated with 10 nM R1881 in the presence of (R)-bicalutamide ((R)-bic), (S)-11 (A) or (R)-9 (B). (TIFF) [file pone.0062657.s002.tiff]

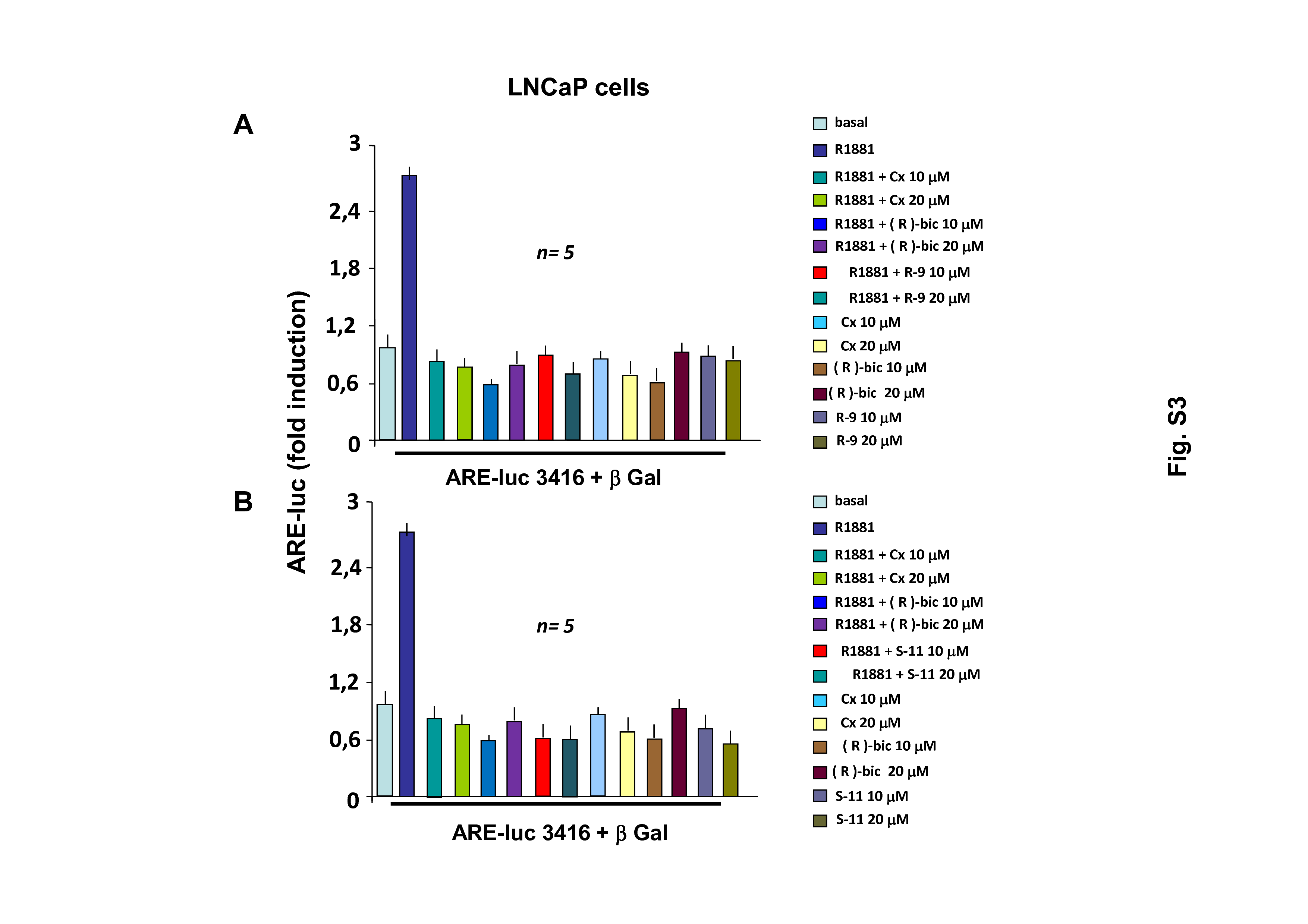

Supplement: Figure S3 — Interference in AR-mediated transcription in LNCaP cells. In A and B, LNCaP cells were transfected with ARE-luc 3416 reporter gene and then made quiescent, as described in the Methods section. Twenty-four hours later, the cells were left untreated (control) or treated for 24 hours with the synthetic androgen R1881 (10 nM) in the absence or presence of the indicated antagonists (used at 10 µM or 20 µM). Luciferase activity was assayed, normalized using β-galactosidase (β -gal) as internal control and expressed as -fold induction. Data from several independent experiments were analyzed. Means and SEMs are shown; n represents the number of experiments. The statistical significance of results in A and B was also evaluated by the paired t test. In both panels, P values were <0.001 for cells stimulated with 10 nM R1881. No significance was attributed to the difference in ARE-luc induction between control cells and cells stimulated with 10 nM R1881 in the presence of bicalutamide (R-bic), (S)-11 (A) or (R)-9 (B). Once again, no significance was attributed to the difference in ARE-luc induction between control cells and cells stimulated with 10 nM R1881 in the presence of Casodex® (Cx ), (S)-11 (A) or (R)-9 (B). (TIFF) [file pone.0062657.s003.tiff]

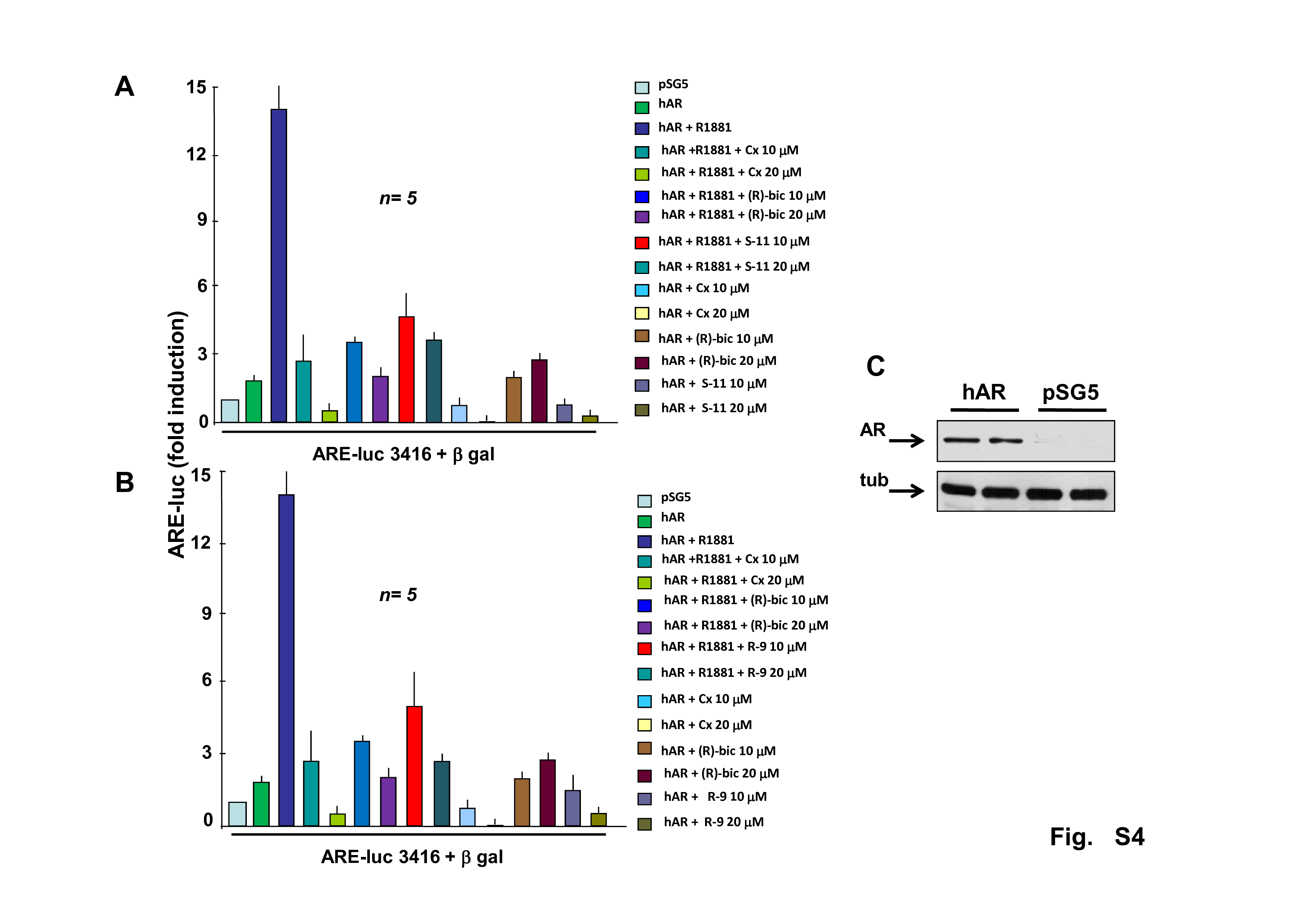

Supplement: Figure S4 — Interference in AR-mediated transcription in Cos-7 cells ectopically expressing hAR. In A and B, AR-negative Cos-7 cells were transfected with pSG5-hAR encoding plasmid together with ARE-luc 3416 plasmid. Control cells were transfected with pSG5 alone. Cells were made quiescent and after 18 hours they were left untreated (control) or treated for 24 hours with the synthetic androgen R1881 (10 nM) in the absence or presence of the indicated antagonists (used at 10 µM or 20 µM). Luciferase activity was assayed, normalized using β-galactosidase (β -gal) as internal control, and expressed as -fold induction. Data from several independent experiments were analyzed. Means and SEMs are shown; n represents the number of experiments. In Cos-7 cells ectopically expressing hAR, the difference in ARE-luc induction between untreated cells and those challenged with 10 nM R1881 was significant (P<0.005 in A and B). In the same cells the difference in ARE-luc induction between the cells stimulated with 10 nM R1881 alone and those stimulated with 10 nM R1881 in the presence of bicalutamide (A and B), (S)-11 (A) or (R)-9 (B) was also significant (P<0.005). In C, lysate proteins were analyzed by Western blot using the antibodies directed against the indicated proteins. AR, androgen receptor. The filter was stripped and re-probed with anti-tubulin antibody (tub) as loading control. (TIFF) [file pone.0062657.s004.tiff]

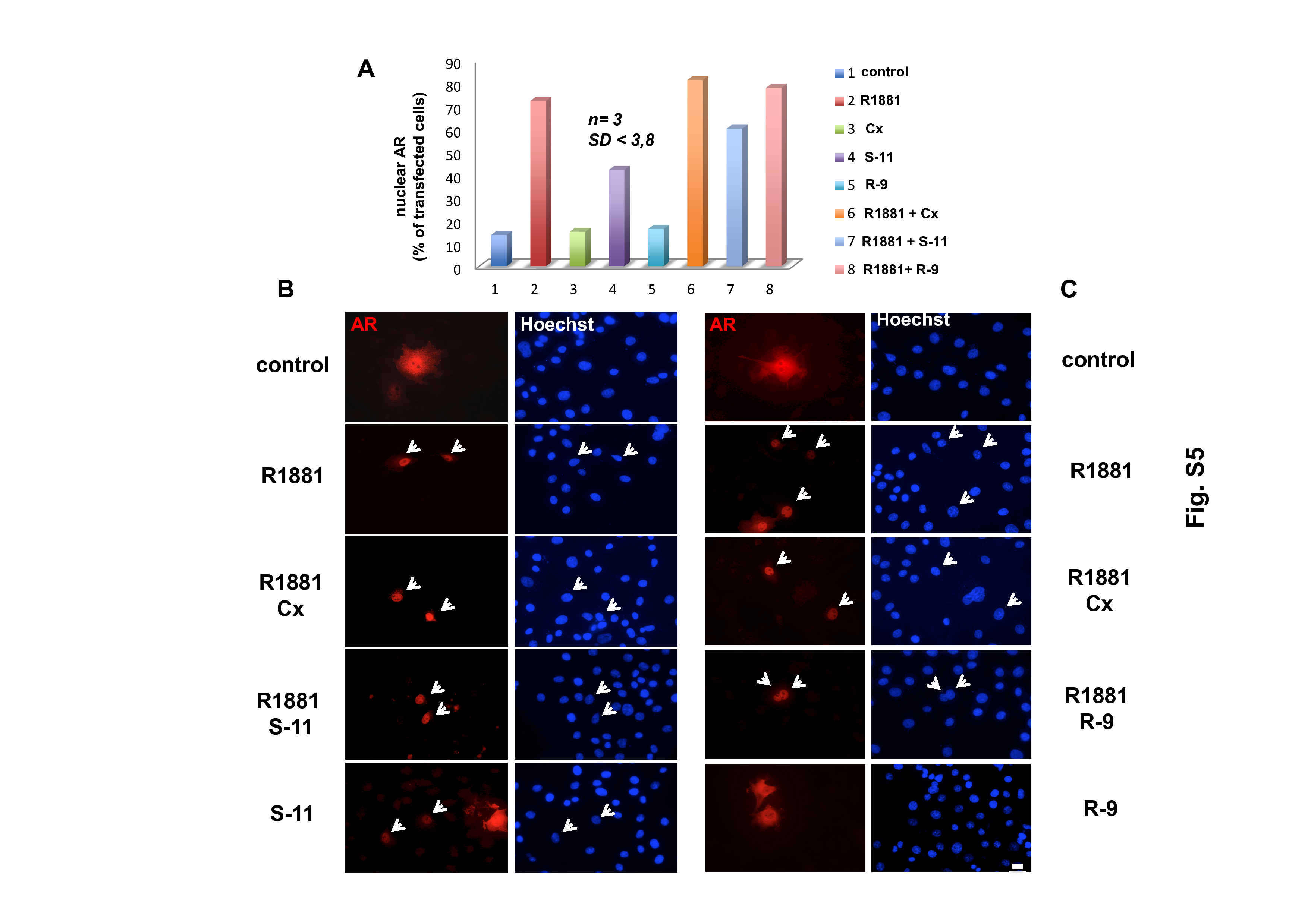

Supplement: Figure S5 — Effect of ( S )-11 and ( R )-9 on nuclear translocation of AR. In A, B and C, AR-negative Cos-7 cells on coverslips were transfected with pSG5-hAR encoding plasmid and then made quiescent. Eighteen hours later, the cells were left untreated (control) or treated for 1 hour with the synthetic androgen R1881 (10 nM) in the absence or presence of 10 µM of the indicated antagonists (Casodex®, Cx). The cells were then analyzed by immunofluorescence for AR, as described in Methods. In A, cells expressing exclusively nuclear AR fluorescence were scored. Results from three different experiments were collected and expressed as % of transfected cells. Data from several independent experiments were analyzed. Means and SEMs are shown; n represents the number of experiments. In Cos-7 cells ectopically expressing hAR, the difference in nuclear AR between the untreated cells and those challenged with 10 nM R1881 was significant (P<0.001). In the same cells the difference in nuclear AR between the un-stimulated cells (control) and those stimulated with 10 nM (S)-11 was also significant (P<0.05). Again, the difference in nuclear AR between the cells challenged with 10 nM R1881 and those stimulated with 10 nM R1881 in the presence of Casodex® or (S)-11 or (R)-9 was not significant. Panels B and C show representative images from one experiment in A. The arrows indicate the cells showing exclusively nuclear AR. Bar, 10 µm. (TIFF) [file pone.0062657.s005.tiff]
