# Supplementary material for: Effect of Small Molecules Modulating Androgen Receptor (SARMs) in Human Prostate Cancer Models
Source: PLoS One. 2013 May 8;8(5):e62657. doi: 10.1371/journal.pone.0062657 (PMC3648536; doi:10.1371/journal.pone.0062657)
Supplement: Table S1 — Antitumor efficacy of antiandrogen on LNCaP and LNCaP-Rbic xenografts. (DOCX) [file pone.0062657.s006.docx]

**Table S1. Antitumor efficacy of antiandrogen on LNCaP and LNCaP-Rbic xenografts**

| **Cell line** | **LNCaP** | | **LNCaP–Rbic** | |
| --- | --- | --- | --- | --- |
| Treatment # | TWI% * | T-C (days)† | TWI (%) | T-C (days) |
| (*R*)-bicalutamide | 50 | 11 | 13 | 2 |
| (*R*)-9 | 66 | 13 | 56 | 16 |
| Casodex® | 47 | 10 | Not done | Not done |

# LNCaP and LNCaP-Rbic cells were injected s.c. into SCID mice at 10^6^ cells/mouse and treatment started on 28^th^ day after tumor cell injection when a tumor weight of 50-150 mg was evident in animals. Drugs were given *per os* at 10 mg/kg/d for 4 consecutive weeks. Five mice *per* group were evaluated. No toxic deaths were observed.

* Percent tumor weight inhibition was calculated at the nadir of the effect. Statistical significance of differences between each group was as follows: LNCaP, (*R*)-bicalutamide *vs* untreated, *P* = 0.001; Casodex® *vs* untreated, *P* = 0.005; (*R*)-**9** vs untreated, *P* < 0.001. LNCaP-Rbic: (*R*)-bicalutamide *vs* untreated, *P* = 0.54; (*R*)-**9** *vs* untreated, *P* = 0.014; (*R*)-**9** vs (*R*)-bicalutamide, *P* = 0.033.

† Calculated as the median times for treated (T) and control (C) tumors to reach the same size (1000 mg). Statistical significance of differences between each group was as follows: LNCaP: (*R*)-bicalutamide *vs* untreated, *P* = 0.009; Casodex® *vs* untreated, *P* = 0.03; (*R*)-**9** vs untreated, *P* = 0.001. LNCaP-Rbic: (*R*)-bicalutamide *vs* untreated, *P* = 0.47; (*R*)-**9** *vs* untreated, *P* = 0.017;

(*R*)-**9** *vs* (*R*)-bicalutamide, *P* = 0.044.
